# The clinical significance of the Neutrophil-to-Lymphocyte Ratio as a novel inflammatory biomarker for assessing the severity of intervertebral disc degeneration

**DOI:** 10.3389/fmed.2024.1446124

**Published:** 2024-10-31

**Authors:** Kai Guo, Jianhua Zeng, Jiawei Lu, Youfeng Guo, Peipei Shan, Yufeng Huang, Desheng Wu

**Affiliations:** Department of Spine Surgery, Shanghai East Hospital, Tongji University School of Medicine, Shanghai, China

**Keywords:** retrospective cohort study, intervertebral disc degeneration, neutrophil-to-lymphocyte ratio, biomarker, systemic inflammation

## Abstract

**Purpose:**

Inflammation is integral to the pathogenesis of intervertebral disc degeneration, yet the role of systemic inflammatory markers in this process remains underexplored. This study aims to explore the association between the Neutrophil-to-Lymphocyte Ratio (NLR) and the severity of disc degeneration.

**Patients and methods:**

A retrospective analysis was conducted on 375 patients diagnosed with lumbar disc degeneration between April 2018 and May 2021. All patients underwent a complete blood cell count examination. We applied the Pfirrmann grading system for cumulative disc grading, stratifying patients into two groups: a high-score group (cumulative grade > 17) and a low-score group (cumulative grade ≤ 17), based on the median cumulative grade. The association between the NLR and and the severity of disc degeneration was further analyzed using correlation analysis and logistic regression models. Furthermore, the predictive capacity of the NLR for lumbar disc degeneration was assessed using the Receiver Operating Characteristic (ROC) curve.

**Results:**

We found a significant positive correlation between high NLR levels and severe disc degeneration. The high-score group exhibited a significantly higher NLR compared to the low-score group [2.63 (1.91–4.18) vs. 2.04 (1.38–2.74), respectively, *p* < 0.001]. Significant correlations were found between NLR and patient characteristics (including age, BMI, VAS, NSAIDs usage, hemoglobin) and the cumulative grading. Logistic regression analysis identified age and NLR as independent predictors of the severity of disc degeneration. The ROC curve analysis demonstrated a good predictive capability of NLR for lumbar disc degeneration.

**Conclusion:**

NLR could serve as a promising biomarker for assessing the severity of lumbar disc degeneration and offer potential benefits in both early diagnosis and treatment strategies.

## Introduction

Intervertebral Disc Degeneration (IDD) is a common disorder and the leading contributor to lower back and discogenic pain, imposing substantial economic costs on both society and families (1). Particularly, Lumbar Disc Degeneration (LDD) is prone to triggering various secondary diseases, including lumbar disc herniation, lumbar spondylolisthesis, and lumbar spinal stenosis, which are major causes of low back pain and neuralgia. Due to degenerative spinal diseases, many patients experience severe pain and functional disorders, leading to further limitations in their mobility, which has emerged as a critical issue in the field of sports medicine. Intervertebral Disc Degeneration (IDD) is a multifaceted process influenced by numerous factors, such as age, lifestyle, mechanical stress, infections, obesity, and genetic predispositions (2, 3). Initiation factors, such as the rupture of the annulus fibrosus and protrusion of the nucleus pulposus, cause morphological changes, a reduction in the number and function of nucleus cells, and loss of major extracellular matrix molecules such as Type II collagen and proteoglycans, which deteriorate the living conditions of the cells (4).

Inflammation could play a crucial role in the incidence, staging, and progression of disc degeneration (2, 5, 6). In patients diagnosed with lumbar degeneration, Neutrophils and lymphocytes are recognized as essential clinical biomarkers for patient stratification and can augment traditional prognostic indicators (7–9). Indeed, heightened local infiltration of immune cells and increased systemic inflammatory responses are potentially critical indicators of the progression and prognosis of disc degeneration (6, 10). Additionally, low-grade chronic inflammation, marked by a persistent elevation of inflammatory cells and pro-inflammatory mediators, frequently manifests prior to the clinical diagnosis of lumbar infection and may hasten the progression of lumbar degeneration. Inflammatory mediators are essential in driving this process (11), with the rapid advancements in molecular biology and immunology, and the expanding research on cytokines and inflammatory mediators, the role of these mediators in disc degeneration is becoming increasingly acknowledged (12). Numerous studies have demonstrated that IVD cells produce significant inflammatory mediators, including interleukin-1, interleukin-8, and interleukin-6, with concentrations notably elevated in degenerated disks (13). Although the precise mechanisms remain to be fully elucidated, these factors are believed to play significant roles in disc degeneration. Inflammation, known to be associated with the onset and progression of many diseases, is characterized by the coordinated activation of various signaling pathways. These pathways regulate the expression of pro-inflammatory and anti-inflammatory mediators in tissue cells. Research indicates that inflammatory factors primarily contribute to disc degeneration by inducing inflammatory responses and promoting cell apoptosis (14). Additionally, the release of pro-inflammatory cytokines facilitates macrophage differentiation and lymphocyte activation, mediating the inflammatory response. This process up-regulates the expression of enzymes that degrade proteoglycans and collagen in the extracellular matrix and leads to metabolic abnormalities in nucleus pulposus cells (15). Within the disc tissue, these inflammatory factors interact, triggering a cascade reaction that exacerbates the inflammatory response and ultimately accelerates disc degeneration.

Systemic inflammation can be assessed by measuring various biochemical or hematological markers in routine blood tests, including specific ratios derived from these indicators. Notably, this includes the Neutrophil-to-Lymphocyte Ratio (NLR), Lymphocyte-to-Monocyte Ratio (LMR), Platelet-to-Lymphocyte Ratio (PLR), and the Systemic Immune-Inflammation Index (SII), which are calculated based on peripheral blood counts of lymphocytes, neutrophils, monocytes, and platelets (16, 17). Previous studies have utilized lymphocytes and monocytes as predictive markers for post-fusion infection, while the LMR has been employed as a predictive biomarker for disc degeneration and lumbar fusion (18, 19). Besides, several studies have also demonstrated a correlation between the NLR and postoperative pain following lumbar spine surgery (20). Significantly, research suggests that the ratios of composite inflammatory cells could offer superior predictive capacity compared to traditional inflammatory markers (21, 22). To enhance understanding of the relationship between the NLR in blood and the severity of disc degeneration, it is essential to evaluate its efficacy as a biomarker for early detection of disc degeneration. Consequently, we conducted a retrospective study to explore how NLR correlates with the severity of lumbar disc degeneration.

## Materials and methods

### Research design

This study retrospectively analyzed 375 patients presenting with lower back pain attributable to lumbar disc herniation or lumbar spinal stenosis, treated at the Department of Spine Surgery Shanghai East hospital between April 2018 and May 2021. In addition to administering questionnaires, multiple physical measurements were taken from participants and a blood sample was collected at the time of admission. This study was conducted in accordance with the Declaration of Helsinki. It was approved by the Ethics Committee of Shanghai East Hospital, and informed consent was obtained from all participants or their legal guardians. Inclusion criteria were as follows: (1) diagnosed with lumbar disc herniation or lumbar spinal stenosis accompanied by severe back pain and radicular pain; (2) presence of radicular symptoms, including pain, sensory abnormalities, and positive straight leg raising test; and (3) imaging data consistent with disc herniation or spinal stenosis at the symptomatic segment as per MRI findings. Exclusion criteria included: (1) lumbar infections, injuries or tumors; (2) previous history of lumbar spine surgery; (3) patients with cauda equina syndrome; (4) patients with a history of chronic diseases such as lung, kidney, and liver diseases; (5) systemic infectious diseases such as osteomyelitis, systemic lupus erythematosus, ankylosing spondylitis, rheumatoid arthritis, etc.; and (6) patients lost to follow-up.

### Data collection

Upon admission, each patient's general baseline information and necessary examination items for the study were collected. These include age, gender, BMI, smoking habits, alcohol abuse history, hypertension, use of non-steroidal anti-inflammatory drugs (NSAIDs), length of hospital stay, neutrophil count, lymphocyte count, and hemoglobin level. The Visual Analog Scale (VAS) was employed to evaluate lower back pain. A VAS score of 0 indicated no pain, scores of 2–4 signified mild pain, scores of 5–7 represented moderate pain, scores of 8–9 denoted severe pain, and a score of 10 indicated the most severe pain. The Pfirrmann grading system, ranging from 1 to 5, was employed to assess the severity of Intervertebral Disc Degeneration (IDD) using magnetic resonance imaging. The classification of disc degeneration are as follows: Grade I: The structure of the disc is homogeneous, with a bright hyperintense white signal intensity and a normal disc height. Grade II: The structure of the disc is inhomogeneous, with a hyperintense white signal. The distinction between nucleus and anulus is clear, and the disc height is normal, with or without horizontal gray bands. Grade III: The structure of the disc is inhomogeneous, with an intermediate gray signal intensity. The distinction between nucleus and anulus is unclear, and the disc height is normal or slightly decreased. Grade IV: The structure of the disc is inhomogeneous, with an hypointense dark gray signal intensity. The distinction between nucleus and anulus is lost, and the disc height is normal or moderately decreased. Grade V: The structure of the disc is inhomogeneous, with a hypointense black signal intensity. The distinction between nucleus and anulus is lost, and the disc space is collapsed (23). The images were independently evaluated in a blinded manner by two experienced spine surgeons. In cases where discrepancies occurred between their assessments, a third senior spine surgeon was consulted to review the images and provide a final consensus. To calculate the cumulative grade, Pfirrmann grades for all lumbar disks were aggregated.

### NLR measured through complete blood count

Blood cell measurements were conducted for all participants. Peripheral blood samples from participants were analyzed within 24 h of collection at the Department of Laboratory Medicine, Shanghai East Hospital, using a Beckman Coulter LH750 Hematology Analyzer. To minimize variability in NLR measurements, all blood samples were collected under standardized conditions. Specifically, blood was drawn between 7:00 a.m. and 9:00 a.m. during routine morning rounds, after an overnight fast. This instrument reported 30 parameters, from which single-cell populations were identified. Specifically, neutrophil, lymphocyte, and monocyte counts were derived as calculated values based on the instrument's differential cell counts. Systemic inflammation marker, Neutrophil-to-Lymphocyte Ratio (NLR), was calculated based on peripheral blood cell counts as NLR = Neutrophils/Lymphocytes.

### Statistical analysis

Statistical analyses were conducted using SPSS version 20.0 software. Participants were divided into two groups, a high-score group and a low-score group, according to their median cumulative scores. The median cumulative grade was determined based on the total score distribution across the entire cohort of patients. The low-score group consisted of individuals with cumulative scores of 17 or less, while the high-score group included those with scores >17. Prior to statistical analysis, the Shapiro-Wilk test was applied to assess the normality of the data. Normally distributed data were represented as mean ± standard deviation (SD), non-normally distributed data were expressed as median [interquartile range], and categorical variables were presented as frequency and *n* (%). Comparisons between groups were conducted using one-way ANOVA and *t*-tests for normally distributed data, the rank-sum test for non-normally distributed data, and chi-square or Fisher's exact test for categorical variables. Multiple comparisons were adjusted using the Bonferroni correction. The Kendall test was employed for analyzing categorical variables, whereas the Spearman test was utilized for continuous variables. Logistic regression analysis was conducted to identify risk factors for severe disc degeneration. Nine multivariate logistic regression models were developed for various indicators, including neutrophils, monocytes, and the NLR. Model fit was evaluated using the Hosmer-Lemeshow goodness-of-fit test, with a Hosmer-Lemeshow statistic ≥ 0.05 indicating an adequate fit. The Receiver Operating Characteristic (ROC) curve was used to evaluate the predictive capability of NLR for severe degeneration, and multiple ROC curves were compared using the DeLong test. The Youden's index was defined as the sum of specificity and sensitivity minus 1. Statistical significance was determined at a two-tailed *p*-value of < 0.05.

## Results

### Baseline characteristics of the patients

The detailed patient characteristics and group comparisons are summarized in Table 1. The average age of the participants was 57.00 years [36.00–69.00] (*p* < 0.001), with 168 females (44.8%). The average BMI of the patients was 23.73 kg/m^2^ [21.63–25.78]. Among the participants, there were 45 cases (12.0%) of smoking, 27 cases (7.2%) of alcohol abuse, 95 cases (25.3%) with hypertension, and 53 cases (14.1%) with diabetes mellitus (DM). A total of 185 cases (49.3%) were using non-steroidal anti-inflammatory drugs (NSAIDs). The median Visual Analog Scale (VAS) score was 5.00 [4.00–6.00], and the median hospital stay was 10.00 days [7.00–13.00]. The median count of neutrophils was 3.97 [3.16–5.34] × 10^9^/L (*p* < 0.005), lymphocyte count was 1.86 ± 0.67 × 10^9^/L (*p* < 0.001), the NLR was 2.27 [1.59–3.35] (*p* = 0.005), and the median hemoglobin (Hb) was 135.00 [124.00–149.00] g/L. Among these variables, age (*p* < 0.001), NLR (*p* = 0.005), neutrophil count (*p* < 0.005), lymphocyte count (*p* < 0.001), and hemoglobin (Hb) (*p* < 0.001) showed significant differences between the high-scoring group (cumulative grade > 17) and the low-scoring group (cumulative grade ≤ 17). The NLR was significantly higher in the high-score group [2.63 (1.91–4.18)] compared to the low-score group [2.04 (1.38–2.74)] (*p* < 0.001). Neutrophil counts were significantly elevated in the high-score group [4.16 (3.40–5.61)] × 10^9^/L vs. the low-score group [3.92 (2.97–4.97)] × 10^9^/L (*p* = 0.005). Lymphocyte counts were significantly lower in the high-score group (1.71 ± 0.71) × 10^9^/L compared to the low-score group (2.01 ± 0.60) × 10^9^/L (*p* < 0.001). However, the distribution of gender, BMI, smoking history, alcohol abuse history, hypertension, DM, NSAIDs usage, VAS scores, and hospital stay did not show significant differences between the two groups. These findings suggest a potential association between higher inflammatory markers and greater severity of disc degeneration.

**Table 1:** Patient characteristics.

**Items**	**All**	**Low score group**	**High score group**	***p***
		**(Cumulative grade** ≤ **17)**	**(Cumulative grade** > **17)**	
Subjects, *n*	375	188	187	
Age	57.00 [36.00–69.00]	39.00 [31.25–58.75]	66.00 [56.00–74.00]	< 0.001
**Gender**				
Male, *n* (%)	168 (44.8)	93 (49.5)	75 (40.1)	0.068
Female, *n* (%)	207 (55.2)	95 (50.5)	112 (59.9)	
BMI, kg/m^2^	23.73 [21.63–25.78]	23.53 [21.34–25.91]	24.14 [21.78–25.77]	0.206
Smoking, *n* (%)	45 (12.0)	25 (13.3)	20 (10.7)	0.438
Alcohol abuse, *n* (%)	27 (7.2)	14 (7.4)	13 (7.0)	0.853
Hypertension, *n* (%)	95 (25.3)	42 (22.3)	53 (28.3)	0.181
DM, *n* (%)	53 (14.1)	22 (11.7)	31 (16.6)	0.175
NSAIDs, *n* (%)	185 (49.3)	85 (45.2)	100 (53.5)	0.110
VAS	5.00 [4.00–6.00]	5.00 [4.00–6.00]	6.00 [4.00–7.00]	0.127
Hospital stay, day	10.00 [7.00–13.00]	10.00 [7.00–12.75]	10.00 [7.00–13.00]	0.520
NLR	2.27 [1.59–3.35]	2.04 [1.38–2.74]	2.63 [1.91–4.18]	< 0.001
Neutrophils, 10^9^/L	3.97 [3.16–5.34]	3.92 [2.97–4.97]	4.16 [3.40–5.61]	0.005
Lymphocyte, 10^9^/L	1.86 ± 0.67	2.01 ± 0.60	1.71 ± 0.71	< 0.001
Hb, g/L	135.00 [124.00–149.00]	138.50 [129.00–152.00]	131.00 [120.00–145.00]	< 0.001

BMI, body mass index; DM, diabetes mellitus; NSAIDs, non-steroidal anti-inflammatory drugs; VAS, visual analog scale; NLR, neutrophil-to-lymphocyte ratio; Hb, hemoglobin.

Normally distributed data were represented as mean ± standard deviation (SD), non-normally distributed data were expressed as median [interquartile range], and categorical variables were presented as frequency and *n* (%).

### Pfirrmann grading distribution and correlation analysis of lumbar disc degeneration

The distribution of lumbar disc degeneration grades, according to the Pfirrmann grading system, is presented in Table 2. Across all participants, the majority of disks in the L1/2, L2/3, and L3/4 levels were classified as Grade 3 and 4. Specifically, at L1/2, 49.1% were Grade 3 and 27.5% were Grade 4. At L2/3, 40% were Grade 3 and 34.7% were Grade 4. At L3/4, 36.5% were Grade 3 and 41.3% were Grade 4. For the L4/5 and L5/S1 levels, a significant proportion of disks were graded as 4 or higher, with 52 and 49.3% of L4/5 disks, and 65.3 and 67.2% of L5/S1 disks, respectively, being classified as Grade 4 or higher. The low-score group exhibited a distribution pattern similar to the overall population. Conversely, with the exception of the L1/2 level, the majority of disc scores in the high-score group were classified as Grade 3 or higher. For individual disks, a Pfirrmann score below 4 typically indicates mild to moderate degeneration, whereas a score of 4 or higher signifies severe degeneration.

**Table 2:** The Pfirrmann grading system for lumbar disc degeneration.

	**1**	**2**	**3**	**4**	**5**
**All (*****n*** = **375)**					
L1/2	7 (1.9)	61 (16.3)	184 (49.1)	103 (27.5)	20 (5.3)
L2/3	7 (1.9)	59 (15.7)	150 (40)	130 (34.7)	29 (7.7)
L3/4	7 (1.9)	46 (12.3)	137 (36.5)	155 (41.3)	30 (8)
L4/5	4 (1.1)	27 (7.2)	92 (24.5)	195 (52)	57 (15.2)
L5/S1	0	107 (28.5)	23 (6.1)	116 (30.9)	129 (34.4)
**Low score group (*****n*** = **188)**					
L1/2	7 (3.7)	57 (30.3)	114 (60.6)	10 (5.3)	0
L2/3	7 (3.7)	58 (30.9)	109 (58)	14 (7.4)	0
L3/4	7 (3.7)	46 (24.5)	104 (55.3)	30 (1)	1 (0.5)
L4/5	4 (2.1)	27 (14.4)	81 (43.1)	70 (37.2)	6 (3.2)
L5/S1	0	75 (39.9)	16 (8.5)	53 (28.2)	44 (23.4)
**High score group (*****n*** = **187)**					
L1/2	0	4 (2.1)	70 (37.4)	93 (49.7)	20 (10.7)
L2/3	0	1 (0.5)	41 (21.9)	116 (62)	29 (15.5)
L3/4	0	0	33 (17.6)	125 (66.8)	29 (15.5)
L4/5	0	0	11 (5.9)	125 (66.8)	51 (27.3)
L5/S1	0	32 (17.1)	7 (3.7)	63 (33.7)	85 (45.5)

Values are expressed as *n* (%).

The analysis of correlations between cumulative disc degeneration grades and patient characteristics (Table 3) revealed significant associations with several factors. Age demonstrated a strong positive correlation (*r* = 0.560, *p* < 0.001), indicating that older patients tend to have higher grades of disc degeneration. Additionally, hypertension (*r* = 0.107, *p* = 0.015) and NSAIDs usage (*r* = 0.101, *p* = 0.022) were positively correlated with higher degeneration grades. Notably, the NLR (*r* = 0.297, *p* < 0.001) and neutrophil count (*r* = 0.210, *p* < 0.001) showed significant positive correlations with cumulative grades, suggesting their potential role in the progression of disc degeneration. In contrast, lymphocyte count (*r* = −0.172, *p* = 0.001) and hemoglobin levels (*r* = −0.198, *p* < 0.001) were negatively correlated with degeneration severity.

**Table 3:** Correlation between cumulative grade and patients' characteristics.

**Item**	***r*/*t***	***P***
Age	0.560	< 0.001
Gender	−0.085	0.053
BMI	0.089	0.086
Smoking	−0.052	0.239
Alcohol abuse	−0.001	0.979
Hypertension	0.107	0.015
DM	0.059	0.177
NSAIDs	0.101	0.022
VAS	0.099	0.056
Hospital stay	0.049	0.343
NLR	0.297	< 0.001
Neutrophils	0.210	< 0.001
Lymphocyte	−0.172	0.001
Hb	−0.198	< 0.001

BMI, body mass index; DM, diabetes mellitus; NSAIDs, non-steroidal anti-inflammatory drugs; VAS, visual analog scale; NLR, neutrophil-to-lymphocyte ratio; Hb, hemoglobin.

Further correlation analysis between NLR and patient characteristics (Table 4) underscored the significance of NLR as an inflammatory marker. NLR was significantly positively correlated with age (*r* = 0.334, *p* < 0.001), BMI (*r* = 0.127, *p* = 0.014), alcohol abuse (*r* = 0.018, *p* = 0.037), and VAS scores (*r* = 0.256, *p* < 0.001), indicating a potential link between higher NLR and increased pain and inflammation. Conversely, NLR was negatively correlated with NSAIDs usage (*r* = −0.196, *p* < 0.001) and hemoglobin levels (*r* = −0.208, *p* < 0.001), suggesting that higher NLR values are associated with lower hemoglobin levels and reduced NSAID consumption. These findings collectively highlight the relevance of NLR and related biomarkers in assessing the severity and progression of disc degeneration, reinforcing their diagnostic and prognostic value.

**Table 4:** Correlation between NLR and patients' characteristics.

**Item**	***r*/*t***	***P***
Age	0.334	< 0.001
Gender	0.068	0.108
BMI	0.127	0.014
Smoking	−0.032	0.452
Alcohol abuse	0.018	0.037
Hypertension	−0.088	0.037
DM	−0.076	0.074
NSAIDs	−0.196	< 0.001
VAS	0.256	< 0.001
Hospital stay	−0.016	0.759
Hb	−0.208	< 0.001

BMI, body mass index; DM, diabetes mellitus; NSAIDs, non-steroidal anti-inflammatory drugs; VAS, visual analog scale; NLR, neutrophil-to-lymphocyte ratio; Hb, hemoglobin.

**Figure 1:**
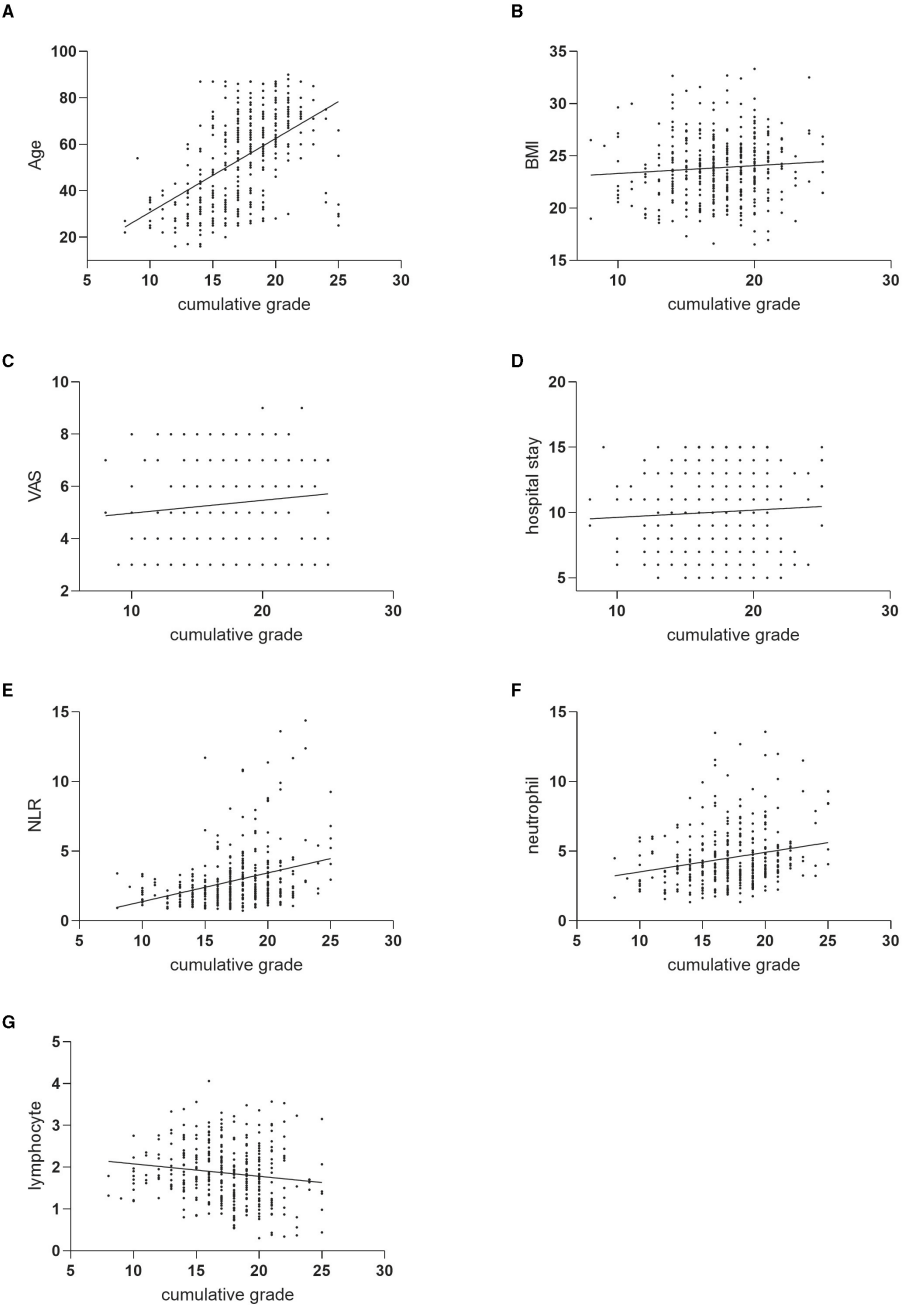
Correlation analysis between cumulative grading and patient characteristics. Scatter diagrams and regression lines for the cumulative grade-age **(A)**, cumulative grade-BMI **(B)**, cumulative grade-VAS **(C)**, cumulative grade-hospital stay **(D)**, cumulative grade-NLR **(E)**, cumulative grade-neutrophil **(F)** and cumulative grade-lymphocyte **(G)** correlation.

Figure 1 illustrates the correlations between cumulative disc degeneration scores and various variables, including age, BMI, VAS scores, hospital stay, NLR, and neutrophil count. The most pronounced correlations were identified with age, NLR, and neutrophil count, indicating that both NLR and neutrophil count are significantly positively correlated with the severity of disc degeneration. These findings suggest that NLR and neutrophil count can serve as valuable indicators for assessing the degree of disc degeneration.

### Analysis of risk factors associated with severe degeneration

**Figure 2:**
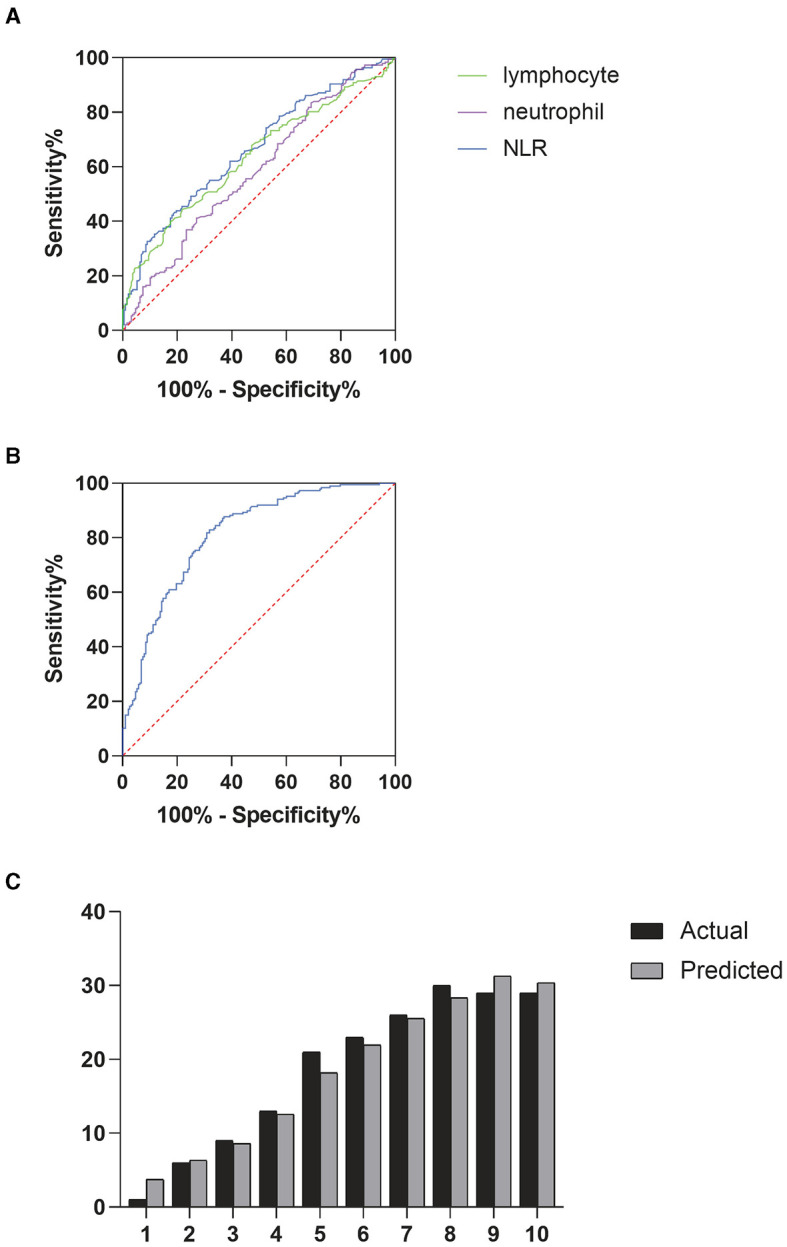
**(A)** Receiver operating characteristic (ROC) curves were used to determine the predictive ability of neutrophils, lymphocytes, and NLR for severe degeneration, **(B)** ROC curve analysis for prognostic model 1, **(C)** deciles of risk for severe degeneration: actual vs. predicted in model 1 (variables shown in Table 6).

Correlation analysis revealed a significant relationship between the cumulative grade and the NLR (*r* = 0.297, *p* < 0.001). A Receiver Operating Characteristic (ROC) curve was then generated to evaluate the predictive capability of NLR for disc degeneration severity. As shown in Figure 2A, the Area Under the Curve (AUC) for NLR was 0.664, higher than that for neutrophils (AUC = 0.585) and lymphocytes (AUC = 0.635), indicating NLR's superior effectiveness in detecting severe disc degeneration. The optimal cut-off value for NLR was 2.99, yielding a Youden's index with a maximum sensitivity of 0.43 and specificity of 0.81, indicating that patients with NLR > 2.99 had a significantly higher likelihood of severe disc degeneration. The AUC for Model 1, illustrated in Figure 2B, was 0.813, suggesting that the model has effective calibration and discrimination abilities. The risk of severe disc degeneration predicted by Model 1 was consistent with the observed risk across risk deciles (see Figure 2C). After adjusting for age and other factors, partial correlation analysis confirmed a significant correlation between NLR and degeneration score (see Table 5, *p* < 0.001). Univariate binary logistic regression identified each unit increase in age (*p* < 0.001), NLR (*p* < 0.001), and hemoglobin (*p* < 0.001) as significantly associated with severe degeneration (see Table 6). Multivariate logistic regression in Model 1, based on clinical parameters, further identified each unit increase in age (OR: 1.071; 95% CI: 1.052–1.091; *p* < 0.001), gender (OR: 0.526; 95% CI: 0.286–0.968; p = 0.039), and NLR (OR: 1.282; 95% CI: 1.093–1.504; *p* = 0.002) as independent predictors of severe disc degeneration.

**Table 5:** Partial correlation between cumulative grade and NLR.

**Control variable**	**Variable**	**Coefficient**	***p*-value**
Age & Gender & BMI & hypertension & Diabetes & Smoking & Alcohol abuse & NSAIDs & Hb	NLR	0.200	< 0.001

BMI, body mass index; DM, diabetes mellitus; NSAIDs, non-steroidal anti-inflammatory drugs; VAS, visual analog scale; NLR, neutrophil-to-lymphocyte ratio; Hb, hemoglobin.

**Table 6:** Univariate and multivariate analysis model 1 of risk factors for IDD.

**Variable**	**Univariate**		**Multivariate**	
	**OR (95% CI)**	***p***	**OR (95% CI)**	***p***
Age	1.066 (1.051–1.081)	< 0.001	1.071 (1.052–1.091)	< 0.001
Gender	0.684 (0.454–1.030)	0.069	0.526 (0.286–0.968)	0.039
BMI	1.036 (0.970–1.107)	0.296	1.015 (0.929–1.110)	0.738
Smoking	0.781 (0.417–1.461)	0.439	0.801 (0.380–1.691)	0.560
Alcohol abuse	0.929 (0.424–2.033)	0.853	1.070 (0.410–2.795)	0.890
Hypertension	1.375 (0.861–2.195)	0.182	0.535 (0.284–1.008)	0.053
DM	1.499 (0.832–2.701)	0.177	0.791 (0.377–1.660)	0.536
NSAIDs	1.393 (0.928–2.091)	0.110	1.210 (0.731–2.004)	0.459
NLR	1.432 (1.237–1.658)	< 0.001	1.282 (1.093–1.504)	0.002
Hb	0.979 (0.968–0.990)	< 0.001	1.014 (0.998–1.030)	0.096

BMI, body mass index; DM, diabetes mellitus; NSAIDs, non-steroidal anti-inflammatory drugs; VAS, visual analog scale; NLR, neutrophil-to-lymphocyte ratio; Hb, hemoglobin.

## Discussion

Intervertebral disc degeneration (IDD) is characterized by a chronic inflammatory process, primarily driven by a significant increase in pro-inflammatory cytokines secreted by IVD cells. These cytokines include tumor necrosis factor, interleukin-1α, interleukin-1β, interleukin-6, and interleukin-17 (13). These cytokines promote the degradation of the extracellular matrix and also stimulate the production of chemokines, leading to changes in the phenotype of IVD cells. This process, leading to an imbalance between catabolic and anabolic reactions, ultimately causes IVD tissue degeneration. Moreover, immune cell recruitment into the disc could be guided by the leakage or fissure-induced exposure of the nucleus pulposus (NP), leading to disc herniation and radicular pain (24). Therefore, intervention strategies aimed at modulating the inflammatory response in degenerative disks may have the potential to prevent or delay the onset and progression of IDD (5). Notably, the intervertebral disc is the largest avascular tissue in the human body, with vascularization confined to a few capillaries present in the cartilage endplate and the outer annulus fibrosus. In the clinical decision-making process, identifying molecular markers could have a significant impact on predicting the outcome of lumbar disc herniation (LDH). These markers could be non-invasive, such as systemic markers, which can provide early warning for clinical decision-making without direct contact with the disc.

Our results indicate that a high NLR is associated with severe lumbar intervertebral disc degeneration. Multivariate analyses have identified a high NLR as an independent risk factor for severe intervertebral disc degeneration. This association is attributed to the pro-inflammatory state, which is a key driver of disc degeneration, potentially accelerating both the onset and progression of the condition (24). Inflammatory cytokines stimulate disc cells to produce chemokines, promoting the recruitment of macrophages, neutrophils, and T cells. Analyses of disc degeneration and herniation have revealed elevated levels of specific chemokines, including monocyte chemotactic protein-1 (MCP-1), CCL3, CCL4, MCP-3, C-X-C motif chemokine 10 (CXCL10), and IL-8 (25, 26), The secretion of cytokines recruits immune cells, initiates an inflammatory cascade, and further exacerbates disc degeneration (27, 28). Research has found that elevated levels of inflammatory cytokines are associated with discogenic low back pain. Additionally, systemic inflammation can influence treatment outcomes and postoperative clinical results in patients with lumbar spine diseases (29). These findings further reveal the central role of inflammatory cytokines and chemokines in recruiting immune cells to the disc and associated tissues, a key step in the pain generation pathway.

In the process of intervertebral disc degeneration, the inflammatory response plays a crucial role. Inflammation can lead to cell damage, cell apoptosis, and matrix alterations, eventually possibly resulting in the degeneration of intervertebral disc tissue. The inflammatory response often involves a variety of cell types, including immune cells such as neutrophils and lymphocytes. Recent research has focused on exploring the response of disc cell-produced cytokines to various environmental stressors and the significance of leukocyte ratios in various inflammatory conditions, including smoking, abnormal mechanical load, cancer, diabetes, injury, and infection (30, 31). Inflammatory indices, such as the NLR and the LMR, are calculated based on the respective counts of neutrophils, lymphocytes, and monocytes. These cells are routinely assessed in most clinical settings globally. Neutrophils and lymphocytes play crucial roles in the inflammatory response. Neutrophils are one of the main cell types in the inflammatory response, capable of releasing inflammatory mediators and enzyme substances such as interleukin-1β, tumor necrosis factor-α, and matrix metalloproteinases. Lymphocytes also participate in the inflammatory response and the process of disc degeneration. Lymphocytes play a crucial role in immune responses, capable of secreting cytokines such as interleukin-6 and tumor necrosis factor-α.

High neutrophil counts and low lymphocyte counts may be associated with inflammation and are independently linked to the prognosis of various diseases (32). For instance, in patients with infections, the most common blood test abnormalities include elevated inflammatory markers such as C-reactive protein (CRP) or erythrocyte sedimentation rate (ESR), high neutrophil counts, and hypoalbuminemia. Multivariable regression models in this study showed that high neutrophil count and low lymphocyte count were independent risk factors for severe degeneration of lumbar disks (Figures 1F, G). Elevated neutrophil counts and reduced lymphocyte counts highlight the potential of NLR as a prognostic biomarker, with higher NLR values indicating greater systemic inflammation. In our research, we uncovered a close relationship between inflammation and intervertebral disc degeneration. In this relationship, the ratios of neutrophils, lymphocytes, and other cell types may play important roles. Furthermore, we found an independent correlation between NLR and the degree of degeneration. Moreover, patients with severe intervertebral disc degeneration exhibited higher NLR values compared to those with mild to moderate degeneration, suggesting a more pronounced systemic inflammatory state. A high NLR indicates elevated local chronic inflammation, which may contribute to the progression of disc degeneration. Simultaneously, in our predictive model, NLR proved to be a highly reliable predictor of severe degeneration. Through the Hosmer-Lemeshow test and ROC curves, we demonstrated that the model had good discrimination and calibration. According to the ROC curve predicting severe degeneration based on NLR, we found that patients with NLR > 2.99 had more severe degeneration. It is noteworthy that although the AUC of NLR in predicting severe disc degeneration in this study did not exceed 0.7, correlation analysis and multivariate regression analysis showed a significant correlation between high NLR and severe disc degeneration. This existing correlation is clinically significant to a certain extent. Similarly, like osteoarthritis, degenerative disc disease is a common comorbidity in obese patients and those with type 2 diabetes. In the pathological states of these two diseases, adipocytokines are important drivers of low-grade inflammation, extracellular matrix degradation, and fibrosis. The accumulation of abnormal fat due to obesity leads to the impairment of white adipose tissue function, which is manifested by the increased production of specific pro-inflammatory proteins (such as adipokines) and cytokines (such as TNF) produced by immune cells in the matrix space. Identifying risk factors for disc degeneration and its progression can help mitigate long-term prognosis and reduce the economic burden on patients, offering significant value for future medical practice.

The results also revealed a significant correlation between the severity of intervertebral disc degeneration and age, hypertension, and NLR. Although lumbar disc degeneration is correlated with low back pain, this study did not find statistically significant results, which might be attributed to the small sample size. Likewise, in addition to NLR, other clinical parameters might affect severe degeneration. Research indicates that disc degeneration increases with age. Although the majority of adults over 30 experience some form of structural degeneration of the intervertebral disc, it doesn't always accompany pain and might represent an aspect of the aging process (33). In fact, many patients with lumbar disc degeneration do not exhibit noticeable symptoms of low back pain in clinical diagnosis. On the other hand, NLR can reflect the inflammatory state in the body and is associated with low back pain, showing significant relations with age, BMI, hypertension, and VAS score. With the progression of degeneration, the levels of inflammatory cytokines will increase, degradation of glycosaminoglycans and collagen will intensify, and changes in the phenotype of intervertebral disc cells will occur (8). According to previous research, through correlation analysis and multiple logistic regression, we found that age is an independent risk factor for severe disc degeneration. This finding further emphasizes the importance of age factors in the prevention and management of disc degeneration.

In summary, the findings of this study reveal the relationship between NLR and the severity of intervertebral disc degeneration, suggesting the potential of NLR as a new biomarker for the early identification of lumbar disc degeneration. This finding also provides new insight into the role of inflammation in disc degeneration and opens new directions for the prevention and treatment of related diseases. However, it's important to acknowledge the limitations of this study. First, the retrospective design may introduce selection bias, as only patients with complete records were included, and information bias, as we relied on pre-existing data. This limits our ability to control for all confounding variables that could influence the results. Additionally, the cross-sectional nature of the data prevents us from evaluating the progression of lumbar disc degeneration and NLR changes over time. A longitudinal study would provide more insight into the dynamic relationship between NLR and disc degeneration and could help determine whether NLR predicts future degeneration. Despite these limitations, based on the results of this study, we can still posit that a single NLR test could be a reliable predictive marker for lumbar disc degeneration. More research is needed in the future to further validate our findings and ascertain their practical clinical implications. Additionally, further investigation into the interplay among different cell types and their relationship with disc degeneration could help us better understand the role of inflammation in disc degeneration and provide new research directions for the prevention and treatment of related diseases. While we are yet to completely overcome the mentioned research limitations. The NLR presents as a promising biomarker for lumbar disc degeneration due to its association with systemic inflammation. As lumbar disc degeneration is often accompanied by a chronic inflammatory state, the NLR can serve as a non-invasive marker to assess the severity of disc degeneration. Clinically, a higher NLR could help identify patients at risk of more severe degeneration and guide therapeutic interventions, especially in earlier stages of the disease.

## Conclusion

This research advances our understanding of the relationship between inflammation and intervertebral disc degeneration, emphasizing the potential of the NLR in blood as a biomarker for identifying and assessing the severity of degenerative disc disease. In summary, NLR could provide clinicians with a valuable tool for assessing the severity of lumbar disc degeneration, offering potential benefits in both early diagnosis and treatment strategies.

## Data Availability

The original contributions presented in the study are included in the article/supplementary material, further inquiries can be directed to the corresponding authors.

## References

[1] Luoma K Riihimäki H Luukkonen R Raininko R Viikari-Juntura E Lamminen A. Low back pain in relation to lumbar disc degeneration. Spine. (2000) 25:487–92. 10.1097/00007632-200002150-00016 10707396

[2] Han CS Hancock MJ Sharma S Sharma S Harris IA Cohen SP. Low back pain of disc, sacroiliac joint, or facet joint origin: a diagnostic accuracy systematic review. EClinicalMedicine. (2023) 59:101960. 10.1016/j.eclinm.2023.101960 37096189 PMC10121397

[3] Kang L Zhang H Jia C Zhang R Shen C. Epigenetic modifications of inflammation in intervertebral disc degeneration. Ageing Res Rev. (2023) 87:101902. 10.1016/j.arr.2023.101902 36871778

[4] Wang SZ Rui YF Lu J Wang C. Cell and molecular biology of intervertebral disc degeneration: current understanding and implications for potential therapeutic strategies. Cell Prolif. (2014) 47:381–90. 10.1111/cpr.12121 25112472 PMC6495969

[5] Cunha C Silva AJ Pereira P Goncalves RM Barbosa MA. The inflammatory response in the regression of lumbar disc herniation. Arthritis Res Ther. (2018) 20:251. 10.1186/s13075-018-1743-4 30400975 PMC6235196

[6] Francisco V Pino J González-Gay MÁ Lago F Karppinen J Tervonen O. A new immunometabolic perspective of intervertebral disc degeneration. Nat Rev Rheumatol. (2022) 18:47–60. 10.1038/s41584-021-00713-z 34845360

[7] Wang L He T Liu J Tai J Wang B Zhang L. Revealing the immune infiltration landscape and identifying diagnostic biomarkers for lumbar disc herniation. Front Immunol. (2021) 12:666355. 10.3389/fimmu.2021.666355 34122424 PMC8190407

[8] Livshits G Kalinkovich A. Hierarchical, imbalanced pro-inflammatory cytokine networks govern the pathogenesis of chronic arthropathies. Osteoarthrit Cartil. (2018) 26:7–17. 10.1016/j.joca.2017.10.013 29074297

[9] Cunha C Teixeira GQ Ribeiro-Machado C Pereira CL Ferreira JR Molinos M. Modulation of the *in vivo* inflammatory response by pro- versus anti-inflammatory intervertebral disc treatments. Int J Mol Sci. (2020) 21:1730. 10.3390/ijms21051730 32138314 PMC7084831

[10] Khan AN Jacobsen HE Khan J Filippi CG Levine M Jr Lehman RA. Inflammatory biomarkers of low back pain and disc degeneration: a review. Ann N Y Acad Sci. (2017) 1410:68–84. 10.1111/nyas.13551 29265416 PMC5744892

[11] Vergroesen PP Kingma I Emanuel KS Hoogendoorn RJ Welting TJ van Royen BJ. Mechanics and biology in intervertebral disc degeneration: a vicious circle. Osteoarthrit Cartil. (2015) 23:1057–70. 10.1016/j.joca.2015.03.028 25827971

[12] Chen X Wang Z Deng R Yan H Liu X Kang R. Intervertebral disc degeneration and inflammatory microenvironment: expression, pathology, and therapeutic strategies. Inflamm Res. (2023) 72:1811–28. 10.1007/s00011-023-01784-2 37665342

[13] Molinos M Almeida CR Caldeira J Cunha C Gonçalves RM Barbosa MA. Inflammation in intervertebral disc degeneration and regeneration. J R Soc Interface. (2015) 12: 20141191. 10.1098/rsif.2014.1191 26040602 PMC4528607

[14] Taniguchi K Karin M. NF-κB, inflammation, immunity and cancer: coming of age. Nat Rev Immunol. (2018) 18:309–24. 10.1038/nri.2017.142 29379212

[15] Kang L Zhang H Jia C Zhang R Shen C. Targeting oxidative stress and inflammation in intervertebral disc degeneration: therapeutic perspectives of phytochemicals. Front Pharmacol. (2022) 13:956355. 10.3389/fphar.2022.956355 35903342 PMC9315394

[16] Dolan RD Lim J McSorley ST Horgan PG McMillan DC. The role of the systemic inflammatory response in predicting outcomes in patients with operable cancer: systematic review and meta-analysis. Sci Rep. (2017) 7:16717. 10.1038/s41598-017-16955-5 29196718 PMC5711862

[17] Hu B Lim J McSoley S Horgan PG McMillan DC. Systemic immune-inflammation index predicts prognosis of patients after curative resection for hepatocellular carcinoma. Clin Cancer Res. (2014) 20:6212–22. 10.1158/1078-0432.CCR-14-0442 25271081

[18] Guo Y Zhao H Lu J Xu H Hu T Wu D. Preoperative lymphocyte to monocyte ratio as a predictive biomarker for disease severity and spinal fusion failure in lumbar degenerative diseases patients undergoing lumbar fusion. J Pain Res. (2022) 15:2879–91. 10.2147/JPR.S379453 36124035 PMC9482412

[19] Iwata E Shigematsu H Koizumi M Nakajima H Okuda A Morimoto Y. Lymphocyte count at 4 days postoperatively and CRP level at 7 days postoperatively: reliable and useful markers for surgical site infection following instrumented spinal fusion. Spine. (2016) 41:1173–8. 10.1097/BRS.0000000000001501 26890955

[20] Bozkurt H Arac D Cigdem B. Effect of preoperative uric acid level and neutrophil/lymphocyte ratio on preoperative and postoperative visual analogue pain scores in patients with lumbar disc herniation: a cross-sectional study. Turk Neurosurg. (2019) 29:705–9. 10.5137/1019-5149.JTN.25897-19.2 30900735

[21] Adamstein NH MacFadyen JG Rose LM Glynn RJ Dey AK Libby P. The neutrophil-lymphocyte ratio and incident atherosclerotic events: analyses from five contemporary randomized trials. Eur Heart J. (2021) 42:896–903. 10.1093/eurheartj/ehaa1034 33417682 PMC7936519

[22] Bhikram T Sandor P. Neutrophil-lymphocyte ratios as inflammatory biomarkers in psychiatric patients. Brain Behav Immun. (2022) 105:237–46. 10.1016/j.bbi.2022.07.006 35839998

[23] Pfirrmann CW Metzdorf A Zanetti M Hodler J Boos N. Magnetic resonance classification of lumbar intervertebral disc degeneration. Spine. (2001) 26:1873–8. 10.1097/00007632-200109010-00011 11568697

[24] Risbud MV Shapiro IM. Role of cytokines in intervertebral disc degeneration: pain and disc content. Nat Rev Rheumatol. (2014) 10:44–56. 10.1038/nrrheum.2013.160 24166242 PMC4151534

[25] Ahn SH Cho YW Ahn MW Jang SH Sohn YK Kim HS. mRNA expression of cytokines and chemokines in herniated lumbar intervertebral discs. Spine. (2002) 27:911–7. 10.1097/00007632-200205010-00005 11979160

[26] Kawaguchi S Yamashita T Katahira G-i Yokozawa H Torigoe T Sato N. Chemokine profile of herniated intervertebral discs infiltrated with monocytes and macrophages. Spine. (2002) 27:1511–6. 10.1097/00007632-200207150-00006 12131709

[27] Lee J Byun H Perikamana SKM Lee S Shin H. Current advances in immunomodulatory biomaterials for bone regeneration. Adv Healthc Mater. (2019) 8:e1801106. 10.1002/adhm.201801106 30328293

[28] Newman H Shih YV Varghese S. Resolution of inflammation in bone regeneration: from understandings to therapeutic applications. Biomaterials. (2021) 277:121114. 10.1016/j.biomaterials.2021.121114 34488119 PMC8545578

[29] Pedersen LM Schistad E Jacobsen LM Røe C Gjerstad J. Serum levels of the pro-inflammatory interleukins 6 (IL-6) and−8 (IL-8) in patients with lumbar radicular pain due to disc herniation: a 12-month prospective study. Brain Behav Immun. (2015) 46:132–6. 10.1016/j.bbi.2015.01.008 25653193

[30] Ma JYQ. Clinicopathological and prognostic significance of lymphocyte to monocyte ratio in patients with gastric cancer: a meta-analysis. Int J Surg. (2018) 50:67–71. 10.1016/j.ijsu.2018.01.002 29329786

[31] Rajan NE Bloom O Maidhof R Stetson N Sherry B Levine M. Toll-Like Receptor 4 (TLR4) expression and stimulation in a model of intervertebral disc inflammation and degeneration. Spine. (2013) 38:1343–51. 10.1097/BRS.0b013e31826b71f4 22850250

[32] Navab M Berliner JA Subbanagounder G Hama S Lusis AJ Castellani LW. HDL and the inflammatory response induced by LDL-derived oxidized phospholipids. Arterioscler Thromb Vasc Biol. (2001) 21:481–8. 10.1161/01.ATV.21.4.481 11304461

[33] Kanayama M Togawa D Takahashi C Terai T Hashimoto T. Cross-sectional magnetic resonance imaging study of lumbar disc degeneration in 200 healthy individuals. J Neurosurg Spine. (2009) 11:501–7. 10.3171/2009.5.SPINE08675 19929349

